# Temporal and Spatial Features of Single-Trial EEG for Brain-Computer Interface

**DOI:** 10.1155/2007/37695

**Published:** 2007-08-30

**Authors:** Qibin Zhao, Liqing Zhang

**Affiliations:** Department of Computer Science and Engineering, Shanghai Jiao Tong University, Shanghai 200240, China

## Abstract

Brain-computer interface (BCI) systems create a novel communication channel from the brain to an output device bypassing conventional motor output pathways of nerves and muscles. Modern BCI technology is essentially based on techniques for the classification of single-trial brain signals. With respect to the topographic patterns of brain rhythm modulations, the common spatial patterns (CSPs) algorithm has been proven to be very useful to produce subject-specific and discriminative spatial filters; but it didn't consider temporal structures of event-related potentials which may be very important for single-trial EEG classification. In this paper, we propose a new framework of feature extraction for classification of hand movement imagery EEG. Computer simulations on real experimental data indicate that independent residual analysis (IRA) method can provide efficient temporal features. Combining IRA features with the CSP method, we obtain the optimal spatial and temporal features with which we achieve the best classification rate. The high classification rate indicates that the proposed method is promising for an EEG-based brain-computer interface.

## 1. INTRODUCTION

Brain-computer interfaces (BCIs) provide new communication and control channels that do not depend on the brain's normal output channels of peripheral nerves and muscles [1]. The BCI research aims at the development of a system that allows direct control of a computer application or a neuroprosthesis, solely by human intentions reflected by certain brain signals [2]. We mainly focus on noninvasive, electroencephalogram- (EEG-) based BCI systems which can be used as tools of communication for the disabled or for healthy subjects who might be interested in exploring a new path of human-machine interfacing.

EEG-based BCI has received increasing attention recently [3–5]. The EEG allows the observation of gross electrical fields of the brain and reflects changes in neural mass activity associated with various mental processes. A physically disabled person with controlling his thoughts has potential to use the mental processes for communication. The feasibility of this communication depends on the extent to which the EEGs associated with these mental processes can be reliably recognized automatically. The electrophysiological phenomena investigated most in the quest for an automatic discrimination of mental states are event-related potential (EP) [3], and localized changes in spectral power of spontaneous EEG related to sensorimotor processes [4, 5]. For noninvasive BCI systems that based on discrimination of voluntarily induced brain states, some approaches have been proposed. The Tübingen thought translation device (TTD) [6] enables subjects to learn self-regulation of slow cortical potentials (SCP), that is, electro cortical positivity and negativity. After training in experiments with vertical cursor movement as feedback navigated by the SCP from central scalp position, patients are able to generate binary decisions in a 4–6 seconds pace with an accuracy of up to 85%. Users of the Albany BCI system [7] are able to control a cursor movement by their oscillatory brain activity into one of two or four possible targets on the computer screen and achieve over 90% hit rates after adapting to the system during many feedback sessions with a selection rate of 4-5 seconds in the binary decision problem. Based on event-related modulations of the pericentral *μ*- or *β*-rhythms of sensorimotor cortices (with a focus on motor preparation and imagination), the Graz BCI system achieved accuracies of over 96% in a ternary classification task with a trial duration of 8 seconds by evaluation of adaptive autoregressive (AAR) models. Note that there are other BCI systems which rely on stimulus/response paradigms, for example, P300, see [2] for an overview. In [8, 9], the common spatial subspace decomposition (CSSD) method was proposed for classification of finger movement and BCI competition 2003-data set IV. The common spatial patterns (CSPs) approach [10, 11] was suggested to be used in the BCI context. This algorithm extracts event-related desynchronization (ERD), that is, event-related attenuations in some frequency bands, for example, *μ*/*β*-rhythm. Further in [12], a first multiclass extension of CSP was presented based on pairwise classification and voting. In this paper, we further extend this approach for extracting both temporal and spatial features of EEG recordings of imaginary left- and right-hand movements. In order to find better features for classification, we use temporal independent component analysis (i.e., IRA) [13] and CSP together for feature extraction. The rest of the paper is organized as follow. In Section 2 , we introduce the neurophysiological background about BCI. In Section 3 , temporal independent component analysis method is derived in detail. In Section 4 , we elaborate the whole procedure of EEG processing including data acquisition, preprocessing, feature extraction, and classification. Finally, classification results are presented and compared with other algorithms.

## 2. NEUROPHYSIOLOGICAL BACKGROUND

**Figure 1:**
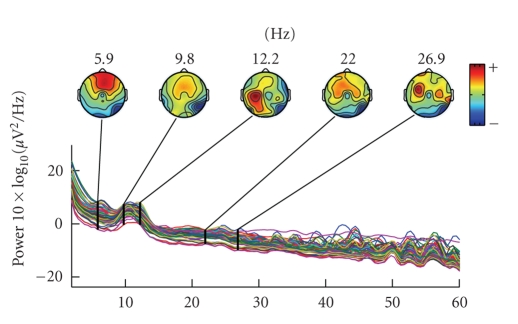
Channel spectra and associated topographical maps during hand movement imagery.

Macroscopic brain activity during resting wakefulness contains distinct “idle” rhythms located over various brain areas, for example, the *μ*-rhythm can be measured over the pericentral sensorimotor cortices in the scalp EEG, usually with a frequency of about 10 Hz. Furthermore, there also exists *β*-rhythm around 20 Hz over the human motor cortex. Therefore, 10 Hz *μ*-rhythm and 20 Hz *β*-rhythm usually coexist in noninvasive scalp EEG recordings (see Figure 1).

As described in [14], each part of the human body exists a corresponding region in the primary motor and primary somatosensory area of the neocortex. The “mapping” from the body part to the respective brain areas approximately preserves topography, that is, neighboring parts of the body are represented in neighboring parts of the cortex. For example, the left hand is represented lateralized on the right hemisphere and the right hand almost symmetrically on the left hemisphere. The temporal amplitude fluctuations of these local rhythms reflect variable functional states of the underlying neuronal cortical networks and can be used for brain-computer interfacing. In particular, the pericentral *μ*- and *β*-rhythms are diminished, or even almost completely blocked by movements of the corresponding body part. Blocking effects are visible bilateral but with a clear predominance contralateral to the moved limb. This attenuation of brain rhythms is termed event-related desynchronization [15].

**Figure 2:**
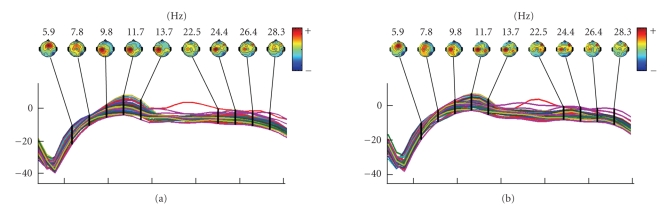
(a) Channels spectra and associated topographical during left-hand movement imagery. (b) Channels spectra and associated topographical during right-hand movement imagery.

**Figure 3:**
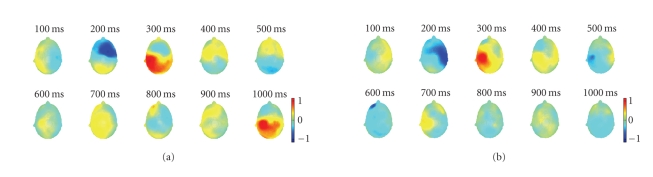
Different temporal amplitude fluctuations and spatial distribution during left- and right-hand movement imagery. (a) A series of 3D scalp maps representing potential distributions at a selected series of time points during left-hand movement imagery. (b) A series of 3D scalp maps representing potential distributions at a selected series of time points during right-hand movement imagery.

Since a focal ERD can be observed over the motor and/or sensory cortex even when a subject is only imagining a movement or sensation in the specific limb, this feature can be used well for BCI control: the discrimination of the imagination of movements of left hand versus right hand can be based on the somatotopic arrangement of the attenuation of the *μ*- and/or *β*-rhythms. Figure 2 shows the average scalp spectra distribution of left hand versus right hand in one trial. The *μ*- and/or *β*-rhythms appeared in both left- and right-hand trials, it is difficult to distinguish them only from frequency spectra of single trial; but they have different characteristics of temporal amplitude fluctuations and spatial distribution (see Figure 3). Therefore, more advanced feature extraction methods should be developed to extract the low diversification of ERD. The CSP algorithm is an effective way to improve the classification performance. There still exists another type of features different from the ERD reflecting imagined or intended movements, the movement-related potentials (MRP), denoting a negative DC shift of the EEG signals in the respective cortical regions. This combination strategy utilizes both temporal and spatial characteristics of EEG data and is able to greatly enhance classification performance in offline studies. In this paper, we focus only on improving the ERD-based classification.

## 3. TEMPORAL INDEPENDENT COMPONENT ANALYSIS

Independent component analysis (ICA) has been accepted as a standard data analysis tool in the neural network and signal processing societies. However, there still exist a number of problems in dealing with real world data using ICA. In many applications, the problem usually does not satisfy the basic assumptions of ICA model. One typical application of ICA is electroencephalographic (EEG) data analysis. EEG usually is very noisy and its mixing model is time-variable. One challenging problem is to extract potential source from single-trial EEG measurements in a very short time window. Still another problem is that ICA generally extract spatial mutual independent source, it did not consider the temporal structures of source signals and then lost the temporal information. Based on that, we suggest to explore both the high-order statistics and temporal structures of source signals. The main idea is to analyze the mutual independence of the residual signals.

### 3.1. Formulation

Assume that **s**(*k*) = [*s*_1_(*k*),*s*_2_(*k*),…*s*_*N*_(*k*)] are mutually spatially independent source signals, of which each temporally correlated with zero mean. Suppose that source *s*_*i*_(*k*) is modelled by a stationary AR model,

(1) $$s_{i}(k)=\sum_{p=1}^{N}a_{p}^{i}s_{i}(k-p)+\varepsilon_{i}(k),$$

where *N* is the degree of the AR model and *ε*_*i*_(*k*) is zero-mean, independently and identically distributed (i.e., white) time series called the residual. For the sake of simplicity, we use the notation $A_{i}(z)=1-\sum_{p=1}^{N}a_{p}^{i}z^{-p},\;z$ is the *z* -transform variable. Since in the blind separation setting the source signals are unknown, we need to impose some constraints on the linear filters. We assume that the linear filters *A*_*i*_(*z*) are minimum phase throughout this paper. Suppose that sensor signals are instantaneous mixtures of the source signals. Let **x**(*k*) = [*x*_1_(*k*),…, *x*_*n*_(*k*)]^*T*^ be the set of linearly mixed signals,

(2) x(k)=Hs(k).

Here, **H** = (*H*_*ij*_) is an *n* × *n* unknown nonsingular mixing matrix. Blind source separation problem is to find a linear transform which transforms the sensor signals into maximally mutually independent components, which are considered as the estimates of source signals. Let **W** be an *n* × *n* nonsingular matrix which transforms the observed signals **x**(*k*) to

(3) y(k)=Wx(k).

The general solution to the blind separation problem is to find a matrix **W** such that **WA** = **ΛP**, where **Λ** ∈ **R** ^*n*×*n*^ is a nonsingular diagonal matrix and **P** ∈ **R** ^*n*×*n*^ is a permutation matrix.

### 3.2. Cost function

In this section, we introduce the mutual information of residual signals as a criterion for training the demixing matrix and temporal structure parameters. The residual independent analysis provides us a new way to explore both the temporal structures and high-order statistics of source signals. From the source model, we have *ε*(*k*) = **A**(*z*)**s**(*k*), where **A**(*z*) can be estimated via the linear prediction method if the source signals **s**(*k*) are known. When the temporal structure **A**(*z*) and the demixing matrix **W** are not well estimated, the residual signals

(4) $$r(k)=(r_{1}(k),\ldots ,r_{n}(k{))}^{T}=A(z)Wx(k)$$

are not mutually independent. Therefore, it provides us a new criterion for training the demixing model and temporal structures to make the residuals **r**(*k*) spatially mutually independent and temporally identically independently distributed.

Assume *q*(**r**) is the probability density function of **r** and *q*_*i*_(*r*_*i*_) is the marginal probability density function of *r*_*i*_, *i* = 1,…, *n*. Now we introduce the mutual information rate *I*(**r**) between a set of stochastic processes *r*_1_,…, *r*_*n*_ as

(5) $$I(r)=-H(r)+\overset{n}{\underset{i=1}{\sum }}H(r_{i}),$$

where *H*(*r*_*i*_) and *H*(**r**) are the entropies of random variables *r*_*i*_ and **r**, respectively. For blind deconvolution problem, Amari [16] and Pham [17] simplify the first term of cost function (5) and derive a cost function as follows:

(6) $$\begin{array}{cc} l(W,A(z)) & =-\frac{1}{2\pi j}\oint_{r}\log\left|det(A(z)W)\right|z^{-1}dz\\ & \;-\frac{1}{L}\overset{L}{\underset{k=1}{\sum }}\overset{n}{\underset{i=1}{\sum }}\log q_{i}(r_{i}(k)), \end{array}$$

where *j* is the imaginary unit of complex numbers, and the path integral is over the unit circle *γ* of the complex plane. The first term of right side of (6) is introduced to prevent the filter **W** from being singular. To simplify the cost function, we calculate the first term of the right side of (6) as follows:

(7) log⁡|det(A(z)W)|=log⁡|det(W)|+log⁡|det(A(z))|.

Because the temporal filters *A* (*z*) is causal and minimum phase, we can easily verify

(8) $$\frac{1}{2\pi j}\oint_{\gamma }\log\left|\text{det}(A(z))\right|z^{-1}dz=0.$$

Now combining equations (7), (8) with (6), we obtain a simplified cost function for independent residual analysis

(9) $$l(W,A(z))=-\log(\left|\text{det}(W)\right|)-\frac{1}{L}\overset{L}{\underset{k=1}{\sum }}\overset{n}{\underset{i=1}{\sum }}\log q_{i}(r_{i}(k)).$$

Independent residual analysis can be formulated into the semiparametric model [18]. The probability density function *q* and the temporal filter **A**(*z*) are seen as the nuisance parameters in the semiparametric model. The demixing matrix **W** is called as the parameters of interest. The semiparametric approach suggests using an estimating function to estimate the parameter of interest, regardless of the nuisance parameters. We suggest to estimate the nuisance parameters in order to have better separating performance of the algorithm.

### 3.3. Conjugate gradient algorithm

In this section, we derive a learning algorithm based on the conjugate gradient descent approach for the demixing matrix. We assume that the probability density functions and the temporal filters are known for a moment during the derivation of a learning algorithm for the demixing matrix. To describe the conjugate gradient method for minimizing cost function, we need first to calculate the natural gradient

(10) $$\nabla l(W,A(z))=(-I+\frac{1}{L}\overset{L}{\underset{k=1}{\sum }}\overset{N}{\underset{p=0}{\sum }}A_{p}[\varphi (r(k))y^{T}(k-p)])W,$$

where *φ* (**r**) = (*φ*_1_(*r*_1_), …*φ*_*n*_(*r*_*n*_))^*T*^is the vector of activation functions, defined by $\varphi_{i}(r_{i})=-{q^{\prime }}_{i}(r_{i})/q_{i}(r_{i})$.

Given an initial value **W**_0_ and *k* = 1, the conjugate gradient algorithm starts out by searching in the steepest descent direction (negative of the gradient) on the first iteration.

(11) $$H_{0}=-\nabla l(W_{0},A(z)).$$

Now we perform one-dimensional search algorithm to find the minimum point of the cost function *l*(**W**, **A**(*z*))

(12) $$W_{k}=\exp(t_{*}H_{k-1}W_{k-1}^{-1})W_{k-1},\;t_{*}=\arg\underset{t}{\min}l(W_{k-1}(t)),$$

along the geodesic: $W_{k-1}(t)=\exp(t_{*}H_{k-1}W_{k-1}^{-1})W_{k-1}$. The new search direction **H**_*k*_ is defined by the following equation:

(13) $$H_{k}=-\nabla l(W_{k})+\gamma_{k}\tau H_{k-1},$$

where *τ* **H**_*k*−1_ is the parallel translation from **W**_*k*−1_ to **W**_*k*_, that is,

(14) $$\tau H_{k-1}=H_{k-1}W_{k-1}^{-1}W_{k}.$$

The value *γ*_*k*_ in (13) is evaluated by

(15) $$\tau_{k}=\frac{\langle G_{k}-\tau G_{k-1},\tau G_{k}\rangle }{\langle \tau G_{k-1},\tau G_{k-1}\rangle }.$$

For the geometrical structures, such as the geodesic and Riemannian metric of nonsingular matrices, refer to [19]. The conjugate gradient algorithm search the minimum point along the geodesic which produces generally faster convergence than steepest descent directions. Both theoretical analysis and computer stimulations show that the conjugate gradient algorithm has much better learning performance than the natural gradient does. Here we briefly introduce learning algorithms for adapting the nuisance parameters in the semiparametric ICA model. By using the gradient descent approach, we obtain the learning algorithm for the filter coefficients $a_{k}^{i}$

(16) $$\text{Δ}a_{p}^{i}(k)=-{\eta^{\prime }}_{k}\frac{1}{L}\overset{L}{\underset{k=1}{\sum }}\varphi_{i}\{r_{i}(k)\}y_{i}(k-p),$$

where ${\eta^{\prime }}_{k}$ is the learning rate. For the detailed information about activation function adaptation, refer to [20].

## 4. METHODS

**Figure 4:**
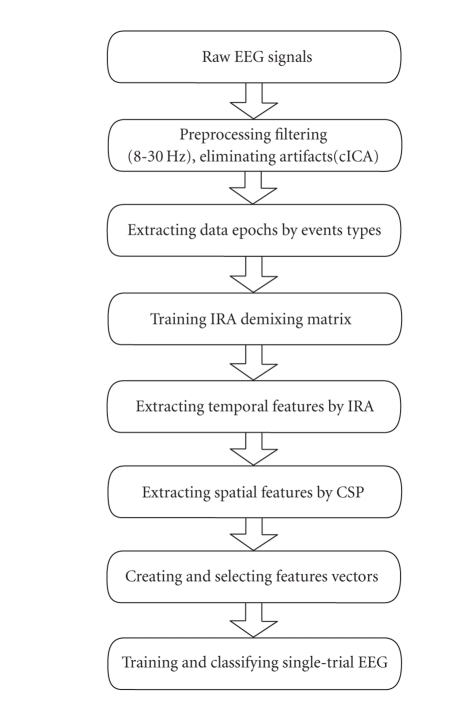
Flowchart of single-trial classification process.

Our procedure to classify the single-trial EEG evoked by left- and right-hand movement imagery is summarized in Figure 4. First, the multichannel EEG signals are preprocessed by cICA method to remove artifacts and/or noise (e.g., EOG). Next, frequency bands (8–30 Hz) are then extracted using band filters, because it mainly contains *μ*- and *β*-rhythm in somatosensory area of the neocortex (see Figure 1). In order to extract both temporal and spatial features of event-related potential, we used combination of IRA and CSP methods followed by a feature selection procedure according to mutual information of each feature and events labels. Finally, two pattern recognition methods of Support Vector Machine (SVM) and linear discrimination analysis (LDA) were carried out, respectively, to give classification results.

### 4.1. Data acquisition

**Figure 5:**
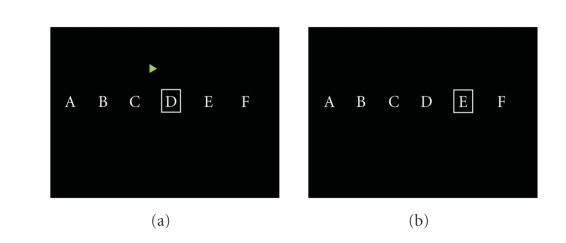
Visual stimulation signals in the experiment paradigm (a) At 3 second, an arrow appeared at the center of the monitor, pointing either to the right or to the left (b) After 4 seconds of imagination, cursor was moved to the next letter.

Our purpose is to develop an online speller paradigm using hand movement imagery EEG to select the letter according to the user's intention. In this paper, we only deal with the offline analysis and test the classification performances of our proposed method. In the experimental sessions used for the present study, labeled trials of brain signals were recorded in the following way: The subjects were seated in an armchair and looked at a computer monitor placed approximately 1.5 m in front at eye level. They were asked to keep their arms and hands relaxed, and to avoid eye movements during the recordings. Each trial started with the presentation of a row of letters at the center of the monitor with cursor on one letter, followed by a short warning tone (“beep”) at 2 second . At 3 second, an arrow appeared at the center of the monitor, pointing either to the right or to the left (“cue”) (Figure 5(a)). Depending on the direction of the arrow, the subject was requested to imagine a movement of the right or the left hand. After 4 seconds, the subject was asked to relax by the “cue” of moving cursor to the next letter towards the direction which the subject imagined (Figure 5(a)). Then next trial began after relaxing for 2 seconds. The experiment comprised six experimental runs of 60 trials in each (30 left and 30 right trials). In the analysis, none of trials was removed for noise.

EEG was recorded referentially from 64 electrodes placed over central and related areas using NeuroScan ESI 128 system at the center for Brain-like Computing and Machine Intelligence, Shanghai Jiao Tong University. The reference electrode was mounted on the left and right mastoids and the grounding electrode on the forehead. The EEG was filtered in a 0.5–200 Hz frequency band. Horizontal and vertical Electrooculogram (HEOG,VEOG) were derived bipolarly using four electrodes. All signals, including 64 channels EEG, EOG, were sampled at 500 Hz. In this study, we use four subjects' experiment data for analysis.

### 4.2. Artifact detection

EEG is often contaminated with ocular and other artifacts. Many methods have been developed in the literature to remove (or attenuate) artifacts in the recordings. Temporally constrained ICA (cICA) [21] can extract signals that are statistically independent, which are constrained to maximizing the correlation with some reference signals. This constraining signal do not need to be a perfect match but it should be enough to point the algorithm in the direction of a particular IC spanning the measurement space.

We assume a set of *k* measured time series **x**(*t*) = [*x*_1_(*t*), *x*_2_(*t*,…, *x*_*k*_(*t*)]^*T*^ to be a linear combination of *l* unknown and statistically independent sources **s**(*t*) = [*s*_1_, *s*_2_,…, *s*_*l*_]^*T*^ (assuming *l* ≤ *k*). A common preprocessing step is to apply a linear “whitening” transformation to the time series so that they have unit variance and are uncorrelated. The cICA is desired to extract a single source of interest and is known as one-unit ICA methods. The natural information-theoretic one-unit contrast function is the negentropy *J* (**y**) :

(17) $$J(y)=H(y_{gaus})-H(y),$$

where *H* (·) is the differential entropy and **y**_gaus_ is a Gaussian random variable with the same variance as the output signal **y**. A more flexible and reliable approximation of negentropy was introduced such that

(18) $$J(y)\approx \rho {\left[E\{G(y)\}-E\{G(v)\}\right]}^{2},$$

where *ρ* is a positive constant, *v* is a zero mean, unit variance Gaussian random variable, and *G*(·) can be any nonquadratic function. The cICA algorithm brings in the use of a constraint which is used to obtain an output which is statistically independent from other sources and is closest to some reference signal **r**(*t*). The closeness constraint can be written as

(19) g(w)=ε(w)−ξ≤0,

where **w** denotes a single demixing weight vector, such that **y** = **w** ^*T*^ **x**; *ε*(**w**) represents the closeness between the estimated output and the reference **r**, and *ξ* represents some closeness threshold. The measure of closeness can take any form, such as mean squared-error (MSE) or correlation, or any other suitable closeness measure. In our implementation of the algorithm, we use correlation as a measure of closeness such that *g* (**w**) becomes

(20) $$g(w)=\xi -E\{r(w^{T}x)\}\leq 0,$$

where *ξ* now becomes the threshold that defines the lower bound of the optimum correlation. With the constraint in place, the cICA problem is formulated as follows:

(21) $$\begin{array}{ll} \text{maximize} & f(w)=\rho {\left[E\{w^{T}x\}-E\{G(v)\}\right]}^{2}\\ \text{Subject to}\; & g(w)\leq 0;\\ & h(w)=E\{y^{2}\}-1=0;\\ & E\{r^{2}\}-1=0; \end{array}$$

where *f* (**w**) denotes the one-unit ICA contrast function, *g* (**w**) is the closeness constraint, *h* (**w**) constrains the output **y** to have unit variance, and the reference signal **r** is also constrained to have unit variance. In [22], the problem of cICA is expressed as a constrained optimization problem which is solved through the use of an augmented Lagrangian function, where learning of the weights and Lagrange parameters is achieved through a Newton-like learning process.

In the field of EEG analysis, it is feasible to assume that some prior information on reference signals is available. In the case of artifact rejection in EEG, the morphologies and relative timings of contaminating eye-blinks or eye-movements can easily be derived in an automated fashion. The relative morphology of the reference is relatively unimportant as long as the temporal features of interest are captured; for example, the use of square “pulses” over the region of interest with a zero reference elsewhere should be reasonable as a temporal constraint when looking for transients such as eye blinks or other similar waveforms. We directly use the channel EOG as a reference function **r**(*t*) to serve as a temporal constraint in the cICA algorithm.

The one-unit cICA method employed for this paper extracts only the single component which is closest to the reference signal in certain sense. However, it is not necessary to assume in advance the number of actual underlying sources, and no manual selection of components is required. These are two very important points for practical implementations of ICA. Generally, the algorithm converges to the desired solution within a small number of iterations and the exact morphology of the reference signal is not too critical in obtaining a plausible solution. This makes it possible for the algorithm to be implemented as an online automatic artifact rejection system. After extracting single component which was regraded as an artifact, we can get the reconstructed noise-free EEG signals by the deflation procedure.

Before feature extraction, the EEG signals are filtered in an 8–30 Hz band. The filter used is a zero-phase forward/backward FIR filter with a width of 20 points. The frequency band was chosen because it encompasses the alpha and beta frequency bands, which have been shown to be most important for movement classification [4]. Furthermore, in a recent movement study, it was shown that a broad frequency band (e.g., 8–30 Hz) gives better classification results compared with narrow bands.

### 4.3. Feature extraction

The IRA is to find the independent source components which also retain temporal structures. These source components can be regarded as different source of neuron electricity and some of them may be related to the motor imagery task. The CSP method is to find a spatial filter according to class labels which maximaize the distance of different class samples. Therefore, theoretically using CSP on IRA components will get better performance than using CSP on mixing signals of EEG. First, we use IRA method to extract some components which mainly contain noise-free EEG components of interest that are of temporal structures. Then CSP will be performed on the components of IRA.

#### 4.3.1. Temporal feature extraction by IRA

**Figure 6:**
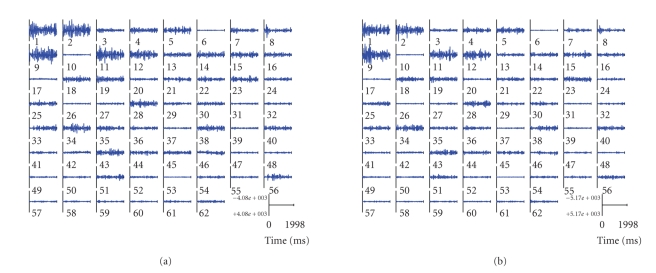
(a) Average 62 components during left-hand movement imagery. (b) Average 62 components during right-hand movement imagery.

Because the temporal structures of event-related potentials may be more obvious after averaging all trials, the IRA was chosen to analyze the averaged source signal obtained from all EEG trials. After the IRA procedure, we obtained separating matrix and source signal sets (see Figure 6). The average-imagined potentials were used for training the IRA demixing matrix which would be used to project the single-trial EEG to IRA bases. The averaged trial can be seen as combination of trials and source components. The common demixing weight matrix will be found by decomposition of averaged trial. After finding the demixing matrix *W*, we will use it for single-trial EEG. In this way, for each movement imagery task, the set of sources signals **s**(*k*) became the features themselves.

**Figure 7:**
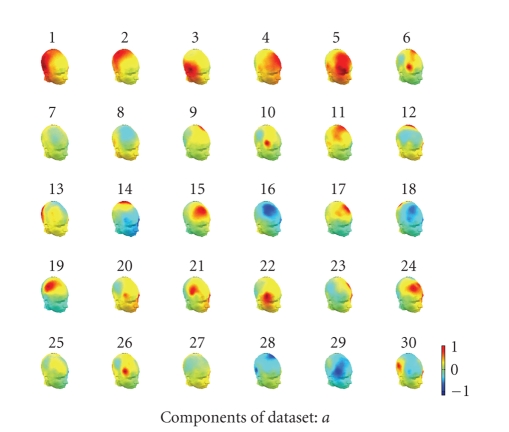
The scalp map projection of the IRA components in 3D head model. Components 9 and 19 were highly related to the motor imagery task, while components 1 and 2 were associated with the occipital alpha rhythm.

According to IRA algorithm, the components are mutually independent, each column in the mixing matrix, represents a spatial map describing the relative projection weights of the corresponding temporal components at each EEG channel. These spatial maps will hereinafter be referred to as IC spatial map. Figure 7 shows 30 IC spatial maps for 30 temporal independent components. In IRA maps, IC9 and IC19 mainly cover left and right motor field of brain which are highly related to the motor imagery task. Therefore, these components can be regarded as source signals that are most effective for classification, which are testified further by mutual information in the Section 4.4 .

#### 4.3.2. Spatial feature extraction by common spatial patterns (CSP)

The common spatial pattern (CSP) algorithm is very useful when calculating spatial filters for detecting ERD effects [23] and for ERD-based BCIs. Given two distributions in a high-dimensional space, the (supervised) CSP algorithm finds directions (i.e., spatial filters) that maximize variance for one class and at the same time minimize variance for the other class. After having band-pass filtered the EEG signals to the rhythms of interest, high variance reflects a strong rhythm and low variance reflects a weak (or attenuated) rhythm.

This criterion is exactly what the CSP algorithm optimizes: maximizing variance for the class of right-hand trials and at the same time minimizing variance for left-hand trials. Moreover, a series of orthogonal filters of both types can be determined. For the analysis, the raw EEG data of a single trial is represented as an *N* × *T* matrix **E**, where *N* is the number of channels (i.e., recording electrodes) and *T* is the number of samples per channel. The normalized spatial covariance of the EEG can be obtained from

(22) $$C=\frac{EE^{\prime }}{\text{trace}(EE^{\prime })},$$

where **E**′ denotes the transpose of *E* and trace(**x**) is the sum of the diagonal elements of **x**. For each of the two distributions to be separated (i.e., left- and right-movement imagery), the spatial covariance ${\bar{C}}_{d\in [l,r]}$ is calculated by averaging over the trials of each group. The composite spatial covariance is given as

(23) $$C_{c}={\overset{¯}{C}}_{l}+{\overset{¯}{C}}_{r}$$

**C**_*c*_ can be factored as $C_{c}=U_{c}\lambda_{c}U{'}_{c}$, where **U**_*c*_ is the matrix of eigenvectors and ***λ***_*c*_ is the diagonal matrix of eigenvalues. Note that throughout this section, the eigenvalues are assumed to be sorted in descending order.

The whitening transformation

(24) $$P=\sqrt{\lambda_{c}^{-1}}U{'}_{c}$$

equalizes the variances in the space spanned by **U**_*c*_, that is, all eigenvalues of **PC**_*c*_ **P**′ are equal to one. If ${\bar{C}}_{l}$ and ${\bar{C}}_{r}$ are transformed as

(25) $$S_{l}=P{\bar{C}}_{l}P',S_{r}=P{\bar{C}}_{r}P'$$

then **S**_*l*_ and **S**_*r*_ share common eigenvectors, that is, if **S**_*l*_ = **Bλ**_*l*_ **B**′ ,then **S**_*r*_ = **Bλ**_*l*_ **B**′ and ***λ***_*l*_ + ***λ***_*r*_ = **I**, where **I** is the identity matrix. Since the sum of two corresponding eigenvalues is always one, the eigenvector with largest eigenvalue for **S**_*l*_ has the smallest eigenvalue for **S**_*r*_ and vice versa. This property makes the eigenvectors **B** useful for classification of the two distributions.

With the projection matrix **W** = **B′ P**, the decomposition (mapping) of a trial is given as

(26) Z=WE.

The columns of **W** ^−1^ are the common spatial patterns and can be seen as time-invariant EEG source distribution vectors. The signals **Z**_*p*_(*p* = 1 ··· 2*m*) that maximize the difference of variance of left versus right-movement imagery EEG are the ones that are associated with the largest eigenvalues *λ*_*l*_ and *λ*_*r*_. These signals are the *m* first and last rows of **Z** due to the calculation of **W**.

#### 4.3.3. Visualization

**Figure 8:**
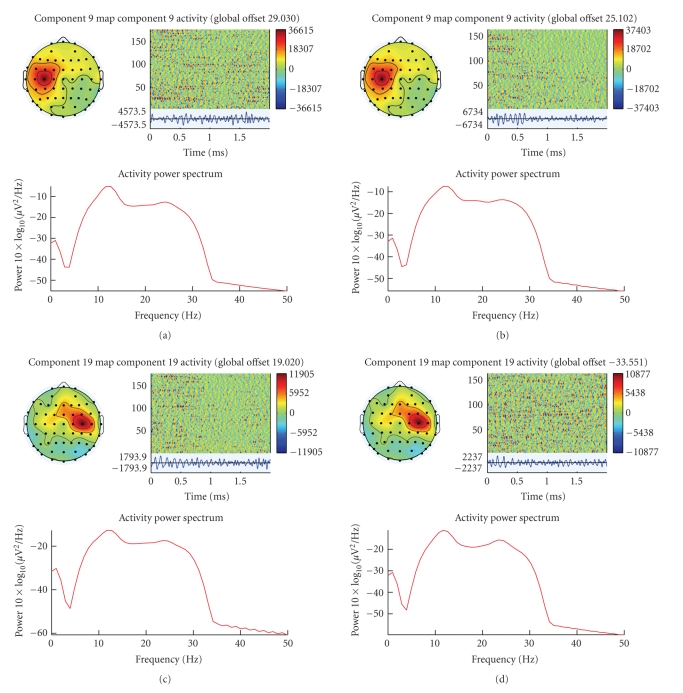
The properties of component which including scalp component map, component activity along trials and power spectrum. (a) Component 9 during left-hand movement imagery. (b) Component 9 during right-hand movement imagery. (c) Component 19 during left-hand movement imagery. (d) Component 19 during right-hand movement imagery. Though the similarity of power spectrum, the temporal amplitude fluctuations of component 9 are obviously different during left- and right-hand movement imagery. In (b), the amplitude has obviously attenuation for all trials while it did not appear in (a).

**Figure 9:**
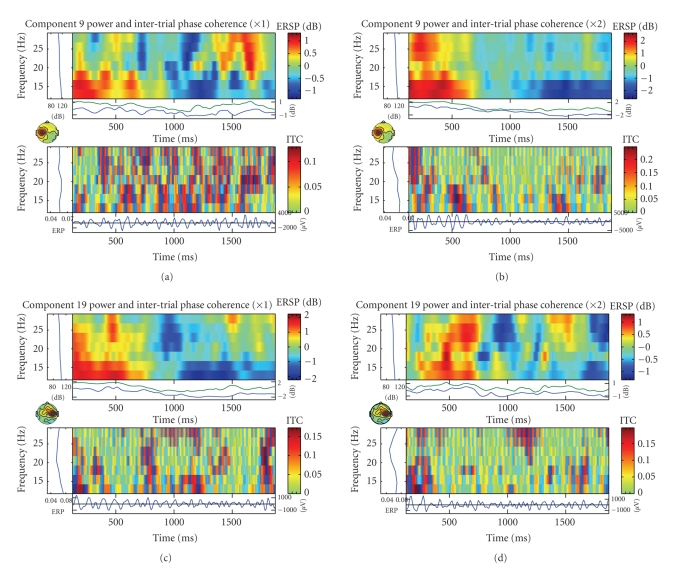
The event-related spectral perturbation (ERSP) shows mean event-related changes in spectral power at each time during the epoch and at each frequency. Intertrial coherence (ITC) indicates degree of that the EEG activity at a given time and frequency in single trials are phase-locked (not phase-random with respect to the time-locking experimental event). (a) ERSP and ITC of component 9 during left-hand movement imagery. (b) ERSP and ITC of component 9 during right-hand movement imagery. (c) ERSP and ITC of component 19 during left-hand movement imagery. (d) ERSP and ITC of component 19 during right-hand movement imagery.

We examine the changes in performance of all trials using a variety of measures and new ideas for visualization that help us to characterize the type and degree of changes seen in EEG features used for BCI classification. We used EEGLAB software package which was an open source toolbox for data visualization. Figure 8 shows components activity along trials and power spectrum. Event-related spectral perturbations (ERSPs) [24] gave each single-trial component activity time series which was transformed to a baseline-normalized spectrographic image using a moving-window average of FFT spectra computed. Intertrial coherence (ITC) is a frequency domain measure of the partial or exact synchronization of activity at a particular latency and frequency to a set of experimental events to which EEG data trials are time locked. The term “inter-trial coherence” refers to its interpretation as the event-related phase coherence (ITPC) or event-related linear coherence (ITLC) between recorded EEG activity and an event-phase indicator function. (See Figure 9.) From ERSP and ITC of components 9 and 19, we found that component 9 of left-hand events and right-hand events has different time-frequency spectral. In left-hand events, featured brief (20–25 Hz) appeared near the middle of the trial, by contrast, right-hand events appeared only near the beginning of the trial. Furthermore, the components 19 of right-hand trials has a little similar time-frequency changes as component 9 of left-hand trials.

### 4.4. Classification

**Figure 10:**
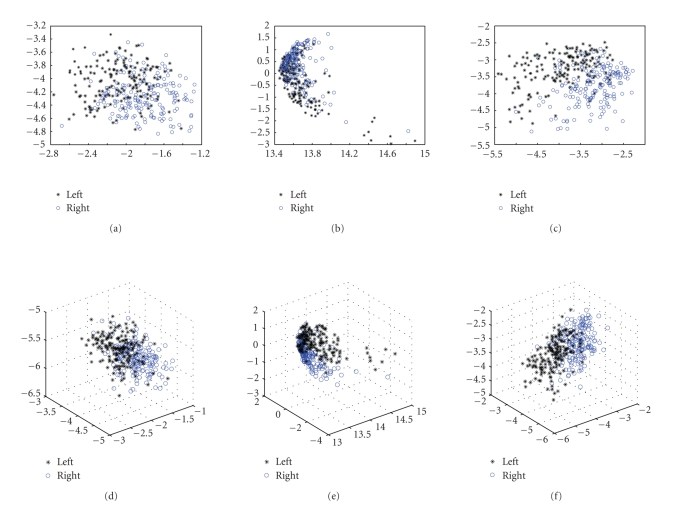
Data distribution of feature vectors in 2-D or 3-D views by using PCA method to reduce dimensions. (a)(d) Feature distribution of two type events which extracted by IRA method. (b)(e) Feature distribution of two type events which extracted by CSP method. (c)(f) Feature distribution of two type events which extracted by our method.

The features used for classification are obtained by IRA and CSP. For each direction-imagined movement, the variances of feature signals suitable for discrimination are used for the construction of the classifier. The feature should maximize the difference of variance of left versus right movement imagery EEG.

(27) $$f_{p}=\log(\frac{\text{var}(Z_{p})}{\overset{n}{\underset{i=1}{\sum }}\text{var}(Z_{i})}),$$

where *Z*_*p*_(*p* = 1 ··· *n* are the CSP components. The feature vectors *f*_*p*_ are used for classification. The log-transformation serves to approximate normal distribution of the data. In order to view the performance of feature extraction methods, we used PCA to reduce feature vectors' dimensions and then viewed ability of separating different classes in 2-D or 3-D space (see Figure 10).

Because some of these features are not sensitive to discriminate different types of single-trial EEG. In fact, there are even irrelevant and redundant features in the feature set. By selecting the relevant features before the classification, we could not only simplify the classifier but also improve the classification performance. The definition of relevant feature is proposed by Blum and Langley [25]. The improved mutual information feature selector (MIFS) algorithm [26] that is chosen in our system for feature selection tries to maximize *I*(*C*; *f*_*i*_ ∣ *f*_*s*_) , and this can be rewritten as

(28) $$I(C;f_{i},f_{s})=I(C;f_{s})+I(C;f_{i}|f_{s}).$$

Here *I*(*C*; *f*_*i*_ ∣ *f*_*s*_) represents the remaining mutual information between class *C* and feature *f*_*i*_ for given *f*_*s*_. For all the candidate features to be selected in the ideal feature selection algorithm, *I*(*C*; *f*_*s*_) is common and not necessary to evaluate it. So the ideal greedy algorithm now tries to find the feature that maximizes *I*(*C*; *f*_*i*_ ∣ *f*_*s*_) (area 3) in (28); but, in general, to calculate *I*(*C*; *f*_*i*_ ∣ *f*_*s*_), we need to divide the input feature space into lots of partitions and this is practically impossible. So we will approximate *I*(*C*; *f*_*i*_ ∣ *f*_*s*_) with *I*(*f*_*s*_; *f*_*i*_) and *I*(*C*; *f*_*i*_), which are relatively easy to calculate. The conditional mutual information *I*(*C*; *f*_*i*_ ∣ *f*_*s*_) can be represented as

(29) $$I(C;f_{i}|f_{s})=I(C;f_{i})-\{I(f_{s};f_{i})-I(f_{s};f_{i}|C)\}.$$

The term *I*(*f*_*s*_; *f*_*i*_ ∣ *C*) means the mutual information between already selected feature *f*_*s*_ and the candidate feature *f*_*i*_ for given class *C*. If conditioning by the class *C* does not change the ratio of the entropy of *f*_*s*_ and the mutual information between *f*_*s*_ and *f*_*i*_ , then the following relation holds:

(30) $$I(f_{s}{;f}_{i}|C)=\frac{H(f_{s}|C)}{H(f_{s})}I(f_{s}{;f}_{i}).$$

Using the equation above and (29) together, we obtain

(31) $$I(f_{i}C|f_{s})=I(f_{i};C)-\frac{I(f_{s};C)}{H(f_{s})}I(f_{s}{;f}_{i}).$$

With this formula, the revised greedy selection algorithm is depicted as follows.

(Greedy selection) repeat until desired number of features are selected.

- (Computation of entropy) for all , *s* ∈ *S*, compute *H*(*s*) if it is not already available.
- (Computation of the MI between variables) for all couples of variables (*f,s*) with *f* ∈ *F*, *s* ∈ *S* compute *I*(*f;s*) if it is not already available.
- (Selection of the next feature) choose feature *f* ∈ *F* as the one that maximizes *I*(*C;f*) − *β* ∑ _*s* ∈ *S*_(*I*(*C;s*)/*H*(*s*))*I*(*f;s*) ; set *F* ← *F*{*f*}, *S* ← {*f*}.

**Figure 11:**
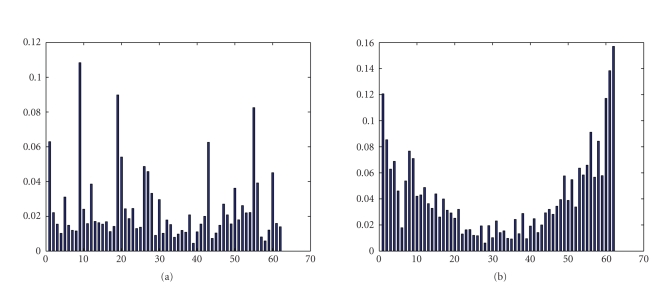
(a) The mutual information of IRA components and events labels. (b) The mutual information of CSP components and events labels.

**Figure 12:**
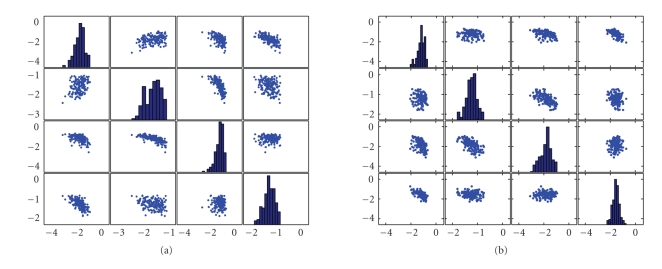
(a) The joint distribution of four features with maximal mutual information between features and events types during left-hand movement imagery. (b) The joint distribution of four features with maximal mutual information between features and events types during right-hand movement imagery.

Here the entropy *H*(*s*) can be computed in the process of computing the mutual information with output class , so there is little change in computational load with respect to MIFS. The variable *β* gives flexibility to the algorithm as in MIFS. If we set *β* zero, the proposed algorithm chooses features in the order of the mutual information with the output. As *β* grows, it deselects the redundant features more efficiently. In general, we can set *β* = 1in compliance with (31). For all the experiments to be discussed later, we set it to 1. The estimation of mutual information (MI) between each feature and event labels are showed in Figure 11. Based on the algorithm, we obtain a subset of relevant features, which possess the larger MI of all the features, for the classification procedure. Figure 12 shows joint distribution of four features with maximal mutual information.

Two classification methods of Support Vector Machine (SVM) and linear discrimination analysis (LDA) were used to validate the result. To evaluate the classification performance, the generalization classification accuracy was estimated by 10-fold cross-validation.

## 5. RESULTS AND DISCUSSIONS

**Figure 13:**
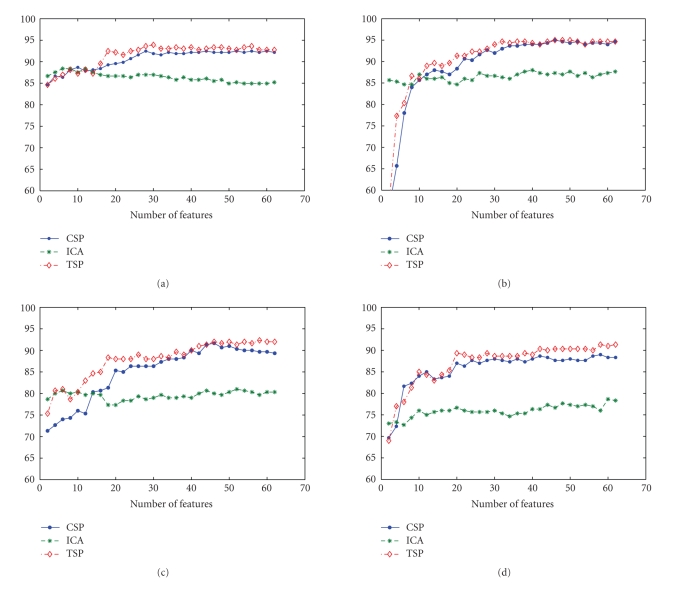
The classification accuracy versus the number of features for CSP, ICA and TSP (combination of IRA and CSP) methods. (a) Subject A. (b) Subject B. (c) Subject C. (d) Subject D.

Table 1 summarizes the results of single-trial EEG classification for left- versus right-hand movement imagery. The first row denotes the different classification method with different number of features, the first column denotes different feature extraction methods for the subjects. In the feature extraction methods, temporal spatial pattern (TSP) represents the method of combining IRA and CSP which we have proposed in this paper. In the table, ICA results are computed by infomax ICA technique through decomposing the data into 62 components and then selecting different number of features based on mutual information method. From the table, we can see that CSP algorithm is sensitive for the frequency (i.e., frequency-specific). ICA results have no obvious improvement with increasing number of features. We also see clearly that the TSP method improves the accuracy of classification. Without applying filtering on EEG signals, TSP method always get better results than the CSP algorithm. Furthermore, Figure 13 shows the curves of classification rate according to number of features. The most optimal result can be obtained by the TSP method and the accuracy is about 93.9% for subject A, 95% for subject B, 92.33% for subject C, and 91.3% for subject D. In the Graz BCI system, subjects were asked to perform the actual finger movement at 8 second and the system also has the feedback to subjects at 1 second after the movement according to the estimate of DSLVQ classifier. However, in our system, the subject only was asked to imagine hand movement but none of actual movement and feedback were performed. In fact, the actual movement will improve the classification rate greatly. Moreover, there is no preselection for artifact trials in our system. Therefore, TSP can provide better features for EEG classification during hand movement imagery and is more suitable for the online BCI system.

The results can be summarized as follows.

1. TSP method (combination of IRA and CSP) can generally increase the classification accuracy of the EEG patterns.
2. CSP is very sensitive to frequency of filtering and is severely subject-specific, while TSP will get better classification rate when dealing with original EEG signals.
3. Temporal features of single-trial EEG which reflects event-related potentials can be used to classify movement imagery tasks.
4. Interrelated feature analysis based on mutual information may improve the EEG classification rate.

## 6. CONCLUSIONS

Single-trial EEG classification is a very difficult and challenging problem in BCI. How to extract effective information or features from original EEG signals becomes a central problem of the EEG-based BCI. In the past BCI research, CSP algorithm has been proven to be very successful in determining spatial filters which extract discriminative brain rhythms. However, the performance can suffer from nondiscriminative brain rhythms with an overlapping frequency range. Meanwhile, IRA algorithm successfully overcomes this problem by finding the latency source related to events. Through IRA decomposition, we will separate useful source components with temporal structures from noise. Therefore, it will overcome the problem of losing temporal information that is very useful for classification of event-related potential. Furthermore, through feature selection based on mutual information, most interrelated or effective features have been selected for classification. It allows to clearly reveal discriminating parts in features set, thus contributes to a better understanding of mechanism for an imagination task. Finally, it would be useful to explore configurations with more than two classes which are more natural and also more friendly from the psychological perspective.

## Figures and Tables

**Table 1:** Classification rates (%) for four subjects with different methods. Denoting temporal spatial pattern (TSP) as method which using both temporal and spatial structure information implemented by IRA and CSP algorithms. The first row denotes different classification method with different number of features and the first column denotes different feature extraction methods for the subjects.

	LDA(4)	SVM(4)	LDA(10)	SVM(10)	LDA(16)	SVM(16)	LDA(24)	SVM(24)	LDA(30)	SVM(30)
Subject A

CSP (no filtering)	71.23	72.78	81.50	82.06	87.14	87.86	87.14	87.86	88.90	89.88
ICA (no filtering)	77.43	77.10	76.69	76.81	76.17	75.94	74.91	74.49	74.17	74.49
TSP (no filtering)	76.55	77.13	87.94	89.01	91.83	92.49	90.02	91.04	90.02	91.04
CSP ([8–30 Hz])	85.48	86.67	87.82	88.69	88.26	88.41	90.96	90.72	91.86	91.88
ICA ([8–30 Hz])	86.85	87.53	86.05	87.53	85.51	86.95	84.06	86.37	83.37	86.95
TSP ([8–30 Hz])	85.56	86.09	85.90	87.24	89.49	89.56	91.96	92.46	93.56	93.90

Subject B

CSP ([8–30 Hz])	65.37	65.66	84.15	85.66	85.86	87.66	90.91	90.33	91.58	92.00
ICA ([8–30 Hz])	85.41	85.33	87.44	87.00	85.97	86.33	86.51	85.66	86.21	86.67
TSP ([8–30 Hz])	76.66	77.33	86.00	86.00	89.00	89.00	92.33	92.33	92.67	94.00

Subject C

CSP ([8–30 Hz])	71.03	72.67	74.70	76.00	79.77	80.67	84.32	86.33	85.25	86.33
ICA ([8–30 Hz])	79.92	80.00	81.42	80.33	79.93	79.67	78.29	78.33	78.04	79.00
TSP ([8–30 Hz])	79.03	80.66	80.90	80.33	86.37	85.00	88.63	88.00	88.14	88.00

Subject D

CSP ([8–30 Hz])	71.89	72.33	82.68	84.00	83.55	83.66	87.82	87.66	86.31	88.00
ICA ([8–30 Hz])	72.63	73.33	74.47	76.00	73.65	76.00	75.31	75.66	75.70	76.00
TSP ([8–30 Hz])	78.01	77.00	84.52	85.00	84.51	84.33	88.27	88.33	88.72	88.66
